# Costs of breast cancer treatment incurred by women in Vietnam

**DOI:** 10.1186/s12889-021-12448-3

**Published:** 2022-01-10

**Authors:** Tran Thu Ngan, Nguyen Bao Ngoc, Hoang Van Minh, Michael Donnelly, Ciaran O’Neill

**Affiliations:** 1 Centre for Public Health, Queen’s University Belfast, Belfast, United Kingdom; 2 Centre for Population Health Sciences, Hanoi University of Public Health, Hanoi, Vietnam

**Keywords:** Breast cancer, Direct cost, Out-of-pocket expenditure, Vietnam

## Abstract

**Background:**

There is a paucity of research on the cost of breast cancer (BC) treatment from the patient’s perspective in Vietnam.

**Methods:**

Individual-level data about out-of-pocket (OOP) expenditures on use of services were collected from women treated for BC (*n* = 202) using an online survey and a face-to-face interview at two tertiary hospitals in 2019. Total expenditures on diagnosis and initial BC treatment were presented in terms of the mean, standard deviation, and range for each type of service use. A generalised linear model (GLM) was used to assess the relationship between total cost and socio-demographic characteristics.

**Results:**

19.3% of respondents had stage 0/I BC, 68.8% had stage II, 9.4% had stage III, none had stage IV. The most expensive OOP elements were targeted therapy with mean cost equal to 649.5 million VND ($28,025) and chemotherapy at 36.5 million VND ($1575). Mean total OOP cost related to diagnosis and initial BC treatment (excluding targeted therapy cost) was 61.8 million VND ($2667). The mean OOP costs among patients with stage II and III BC were, respectively, 66 and 148% higher than stage 0/I.

**Conclusions:**

BC patients in Vietnam incur significant OOP costs. The cost of BC treatment was driven by the use of therapies and presentation stage at diagnosis. It is likely that OOP costs of BC patients would be reduced by earlier detection through raised awareness and screening programmes and by providing a higher insurance reimbursement rate for targeted therapy.

**Supplementary Information:**

The online version contains supplementary material available at 10.1186/s12889-021-12448-3.

## Introduction

Healthcare services including cancer treatment in Vietnam are financed via three main sources: government budget, social health insurance (SHI), and out-of-pocket (OOP) payments [1, 2]. The largest share of total health expenditure is OOP which increased from 37% of total health expenditures in 2011 to approximately 45% in 2017 [1, 3]. Vietnam was ranked 46/186 countries in terms of the share of health expenditure from OOP which was higher than the 30% upper bound recommended by the World Health Organization (WHO) [2, 3]. The proportion of Vietnamese households who suffered ‘catastrophic’ health expenditure (CHE) due to OOP payment (i.e., household’s total OOP payment is equal or exceed 40% of household’s income) was 4–6% during the 2000s and rose to 10% in 2016 [2, 4]. However, CHE among households with cancer patients was as high as 65% [5].

Breast cancer (BC) is the most common cancer among Vietnamese women with increasing incidence over time and the highest number of cancer-related deaths [6, 7]. CHE occurred more often among households with breast cancer patients compared with households of patients with other cancer types (72% vs 57%, respectively) [5]. Previous studies showed that high OOP payment was associated with medication adherence, service utilisation, and treatment outcomes [8–10]. However, the level and composition of OOP payment related to BC treatment in Vietnam and the drivers of expenditure remain largely unexamined. The only study to examine the cost of BC care in Vietnam assessed the direct medical cost of treatment by category and stage of cancer [11]. However, the representativeness and currency of the data which was from 2001-2006 for only one tertiary hospital in central Vietnam call into question its relevance. Moreover, as it was confined to an examination of cost from the public payer’s perspective (i.e., SHI and the government) [11], it offers limited insight into costs incurred by patients.

The treatment and timing/dosage of drugs depend on the stage and the pathology type of breast cancer [12]. Ideally, treatment is ‘personalised’ so that the optimum result with the lowest toxicity and the least undesirable effects is achieved [12]. Thus, using patient-level data in the calculation of costs (rather than calculating according to clinical guideline-informed treatment pathways) can reveal between-patient heterogeneity [13]. In other words, patient-level costing allows the comparison of costs between subgroups (defined by stage at diagnosis and treatment options) as well as the identification of cost drivers. The transferability of estimates from other jurisdictions are open to question [10, 13]. In this context, there is a need to gather data about current costs from a patient perspective to understand service uptake and the financial burden of care. This study sought to address this gap in the literature by using patient-level data to assess the OOP expenses regarding diagnosis and initial treatment (D&T) of Vietnamese BC patients by stage and category of treatment.

### Social health insurance system and breast cancer care in Vietnam

SHI is currently regarded as the main method of public financing for healthcare in Vietnam [14]. It was established in 1992 and became mandated for everybody since 2014 [15, 16]. However, due to inadequate monitoring and inspection as well as enforcement, the population coverage stood at 81.7% in 2016 [7, 14]. When patients use healthcare services included in the benefit package of SHI, the insurer will cover 80% (most common), 95%, or 100% of the total service cost (i.e., reimbursement rate) while the remaining 20, 5, and 0%, respectively, are met by co-payments incurred by patients [16]. The poor, military personnel, ethnic minorities, and war veterans are eligible for 0% co-payment while students/pupils, retired persons, and members of near-poor households are eligible for co-payment at 5% [16].

Most of healthcare services and drugs for BC is included in the benefit package of SHI except Trastuzumab (SHI covers 48–60% of the cost, depends on the reimbursement rate the insured is entitled to), breast reconstruction surgery, and Pertuzumab (both are not covered). However, healthcare providers are allowed to sell services not covered by SHI (i.e., service-on-demand) and clinicians can prescribe drugs that are not covered by SHI [14, 15]. Financial autonomy of hospitals and provider payments based on fee-for-service scheme can lead to ‘induced demand’ by providers in the types of overprescribe drugs, overprescribe diagnosis tests plus test repetition when patients move from a hospital to another, and prolong in-patient stay. These, in turn, can lead to higher OOP payment for patients [17, 18].

## Methods

### Study design and participants

We analysed data from a 2019 study of medical expenses and health-related quality of life of women with BC through an online survey and a hospital-based face-to-face survey. The online survey recruited participants from all over the country (response rate was 69%) while the hospital-based survey was conducted in two tertiary hospitals (response rate was 99%). Participants were BC patients/survivors with no restriction regarding demographic characteristics such as age, ethnic group, education, occupation, and residence area. Data collection was restricted to 3 months for the online survey and 3 days for the hospital-based surveys considering available resources and logistics of conducting face-to-face interviews at hospitals. The resulting sample comprised a higher proportion of individuals with higher education level and from urban areas if compared with national data. Details about study design and methodology are available elsewhere [19].

Following diagnosis, BC treatment in Vietnam has two main courses including the ‘initial treatment’ and the ‘follow-up treatment’ [11, 12]. The former starts right after the diagnosis and often lasts up to 9 months. It includes surgery (e.g., lumpectomy, mastectomy, breast reconstruction surgery), radiotherapy, and systemic therapies (e.g., chemotherapy, hormone therapy, targeted therapy) [11, 12]. After initial treatment, follow-up treatment including hormone therapy (for those are eligible to prevent recurrence of BC) and periodic check-up may last 5–10 years with outpatient appointments every 3–6 months [11, 12]. Participants of the original study included both women who were receiving ‘initial treatment’ and those who had finished initial treatment and were discharged from hospital. Costs of follow-up treatment is difficult to precisely estimate given recall bias and unknown compliance [11] and costs of initial treatment were unknown for those had not yet completed this treatment. Therefore, we only analyse the costs of D&T from those had already finished the initial treatment.

### Assessment of costs

This study presents patient-reported direct payment to health care providers at time-of-service use (excluding the amount covered by SHI, if any) which is the costs of BC D&T from a patient perspective. Mean costs for each health service were calculated among those who utilised that corresponding service. All costs were converted to 2019 prices using a Gross Domestic Product deflator index for Vietnam [20]. Costs were presented in Vietnamese Dong (VND) and US dollars (USD) for comparative purposes. The exchange rate used was 1 USD = 23,176 VND recorded on November 12, 2020.

#### Cost of diagnosis

Health services related to diagnosis included clinical diagnosis (e.g., clinical breast examination and medical history asking), laboratory diagnosis (e.g., mammography, ultrasound, magnetic resonance imaging-MRI, nuclear medicine imaging), and histopathological diagnosis (e.g., fine needle aspiration-FNA, core needle biopsy, vacuum-assisted breast biopsy-VABB) [12]. Patients may use these services several times at different hospitals before starting the treatment [21]. Therefore, respondents reported the lump sum of all health services related to diagnosis that they received rather than the cost of each individual service by answering the question ‘What were the costs you paid for health services related to examination and diagnosis?’

#### Cost of initial treatment

Respondents identified the treatments that they received from a list including lumpectomy, mastectomy, breast reconstruction surgery, chemotherapy, radiotherapy, hormone therapy, and targeted therapy. For each of the utilised services, respondents reported how much was the OOP expense by answering the question ‘What were the costs you paid for that service?’. For treatments that respondents did not utilise, the costs were recorded as zero. Questions used to estimate the cost of BC D&T are presented in the supplementary material.

#### Covariates

The choice of covariates that were used to explain variation in costs was based on previous studies [11, 13]: stage of cancer at diagnosis, SHI reimbursement rate, age, household monthly income, and education level. Respondents were asked ‘What was their stage of cancer at diagnosis’ with simplified options of ‘stage 0’, ‘stage I’, ‘stage II’, ‘stage III’, ‘stage IV’, and ‘do not know/do not remember’ to minimise recall bias. Age of respondents was recorded in years while the SHI reimbursement rate was recorded as percentage of health services’ total cost that will be paid by the insurer (i.e., 80, 95, 100%). Total households’ monthly income (both formal and informal) had five categories ‘≤ 3,000,000 VND’, ‘3,000,0001-6,000,000 VND’, ‘6,000,0001-9,000,000 VND’, ‘9,000,001–12,000,000 VND’, and ‘> 12,000,000 VND’ based on income quintiles of general Vietnamese households in 2016 [22].

### Data analysis

Descriptive statistics (mean and standard deviation-SD for continuous variables, percentages for discrete variables) were used to describe the sociodemographic and clinical-related characteristics of respondents. Costs were presented with and without outliers. Outliers were defined as values which fell more than 1.5 times the interquartile range (IQR) above the third quartile (Q3) or below the first quartile (Q1) (outliers if value > Q3 + 1.5IQR or < Q1–1.5IQR) [23].

Apart from cost analysis by type of treatment, total cost of D&T of BC was analysed by respondent key characteristics using a generalised linear model (GLM). Total cost was calculated by summing diagnosis and treatment costs excluding the cost of targeted therapy. In Vietnam, targeted therapy is optional and patients choose between usual chemotherapy or targeted therapy based on their perceived need and ability to pay. Due to its extremely high cost and low reimbursement rate from SHI (48–60% for Trastuzumab, 0% for Pertuzumab [24]), the cost of targeted therapy alone was more than ten times higher than the total cost of diagnosis and all other treatment types. Therefore, targeted therapy cost was excluded in the modelling of the associated factors of total cost.

Component costs contained outliers and missing data (6–66% depending on the specific treatment concerned). To avoid biased parameter estimates if applying listwise deletion or complete-case analysis for this large number of missing observations [25, 26], we used a multiple imputation technique to impute missing values. Prior to the modelling, outliers were recoded as missing and then all missing data was imputed using the multivariate imputation by chained equations (MICE) method where all component costs are imputed sequentially [25, 26]. While a number of imputations (M) = 5 often deemed sufficient [26, 27] we chose M = 20 to increase the stability of the results. As the cost data was skewed and bounded by specific value (cost = 0 if the service were not used and no cost could be negative), we used the recommended method: predictive mean matching (PMM) with condition to predict the imputed value [25, 26, 28]. PMM randomly draws an imputed value from a set in the donor pool (observations with the closest value predicted by linear regression model for the missing one) [25, 26, 28]. The condition was that imputed values would be given only for those who used the treatment type; otherwise, missing values would be replaced with zero. The total cost was calculated after this imputation procedure.

## Results

Respondent characteristics are presented in Table 1. The mean age (SD) of respondents was 48.4 (10.1) years, 78% were married, 70% were working full-time or self-employed, and 87% completed at least high school education; 88% were diagnosed at early stage (stage 0/I/II), 10% were diagnosed at stage III and none at stage IV; all respondents possessed SHI and 82% had SHI with 80% reimbursement rate.

**Table 1:** Characteristics of sample

Characteristics (***n*** = 202)	Number	Percentage
Age (in years), mean (SD)	48.4 (10.1)	
Education level		
Completed at least secondary education	27	13.4
Completed high school education	38	18.9
Completed undergraduate	122	60.7
Completed graduate	14	7.0
Marital status		
Single/separated/divorce/widow	44	22.0
Married	156	78.0
Occupation		
Unemployed/Student/Homemaker	23	11.6
Full-time employee	96	48.2
Self-employed	44	22.1
Retired	36	18.1
Household income (in Vietnamese Dong – VND)		
≤ 3,000,000 VND (~$129)	18	9.2
3,000,001–6,000,000 VND ($130–259)	33	16.9
6,000,001–9,000,000 VND ($260–389)	20	10.3
9,000,001–12,000,000 VND ($390–519)	55	28.2
> 12,000,000 VND ($519)	69	35.4
Stage of cancer at diagnosis		
Stage 0/I	39	19.3
Stage II	139	68.8
Stage III	19	9.4
Don’t know/Don’t remember	5	2.5
Reimbursement rate of social health insurance		
80%	160	81.6
95%	15	7.7
100%	21	10.7

*VND* Vietnamese Dong (the currency of Vietnam) | $: United States Dollar (USD)

Exchange rate in November 2020: 1 USD = 23,176 VND

**Fig. 1:**
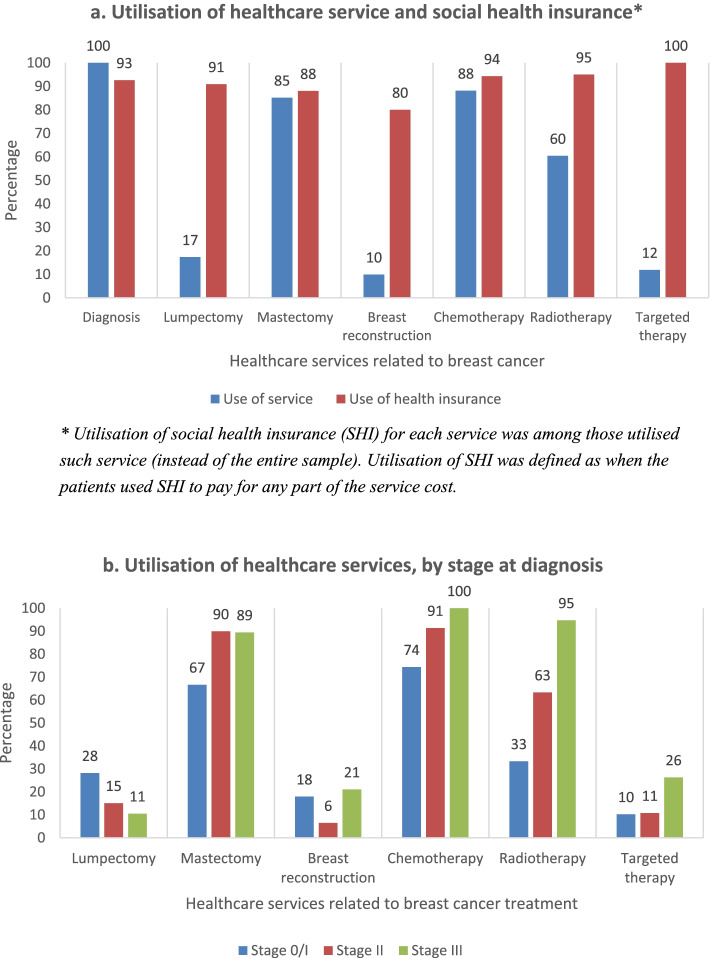
Utilisation of health services (related to breast cancer) and social health insurance

Health service utilisation (related to breast cancer) and the corresponding utilisation of SHI to pay for any part of the cost, among respondents who used such services are presented in Fig. 1. The most used treatment therapies were chemotherapy (88%), mastectomy (85%), and radiotherapy (60%). Only 10–12% of respondents had breast reconstruction surgery and targeted therapy, respectively. The use of SHI was higher than 80% for all types of services. The lowest utilisation of SHI was for breast reconstruction surgery (80%) and mastectomy (88%). All respondents (100%) used SHI to pay for targeted therapy cost. Compared to stage 0/I, the proportion of patients in stage II/III that utilised mastectomy, chemotherapy, and radiotherapy was significantly higher (Fig. 1b; chi-square test, *p* < 0.001; test not shown).

**Fig. 2:**
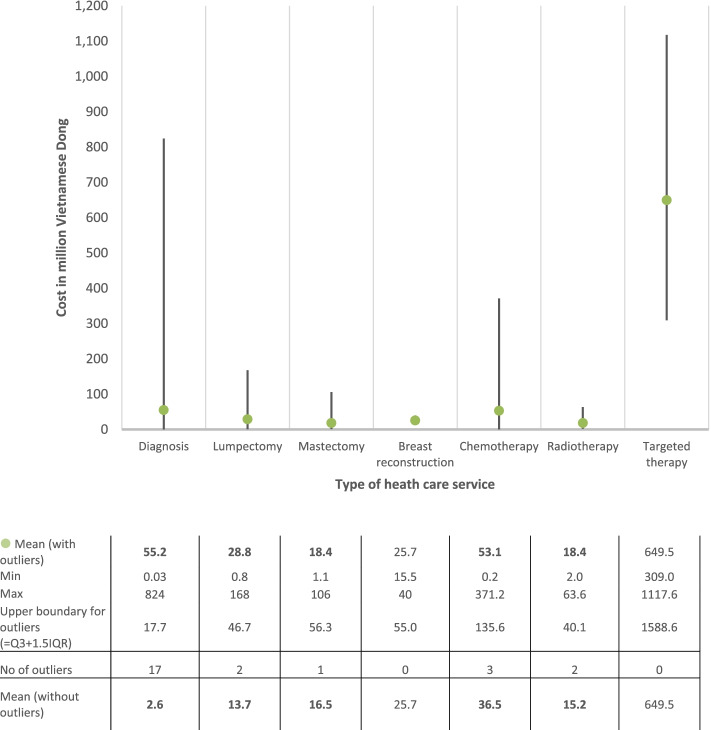
Variation of costs in breast cancer diagnosis and initial treatment

Figure 2 shows variation in costs for each category of BC D&T among those who used the corresponding services. The costliest service was targeted therapy with mean cost at 649.5 million VND (~$28,025). Diagnostic costs had the widest range from 0.03 to 824 million VND ($1.3–35,554) due to the highest number of outliers (17 outliers). When the outliers were removed, diagnosis was the cheapest service with mean cost at 2.6 million VND ($112). The second most costly and skewed cost was chemotherapy with range from 0.2 to 371.2 million VND ($8.6–16,017) and mean (without outliers) at 36.5 million VND ($1575).

**Fig. 3:**
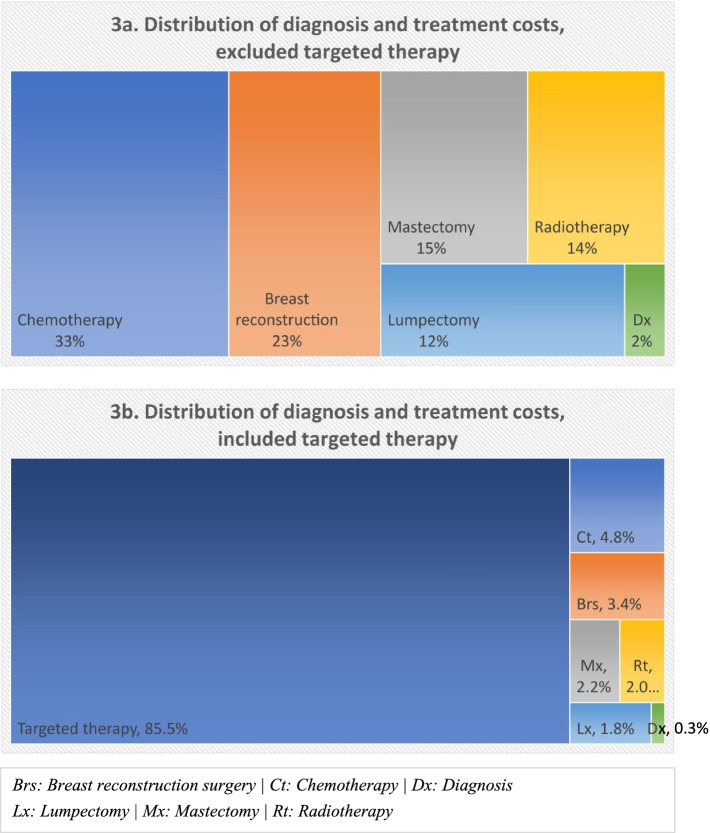
Distribution of breast cancer diagnosis and initial treatment costs

Figure 3 shows the costs of each BC D&T type in relation to other services’ cost and the total cost, with and without targeted therapy (Fig. 3b and a, respectively). When targeted therapy was not included, chemotherapy and breast reconstruction surgery made up of approximately half the total cost (33 and 23%, respectively). The cost of lumpectomy, mastectomy, and radiotherapy shared 12–15% of the total cost while diagnosis cost only accounted for 2%. When targeted therapy was included into the treatment regime, it made up the majority of treatment cost (85.5%).

GLM results showed that later stage of cancer diagnosis and higher education level of respondents were associated with the higher total cost for breast cancer (Table 2). Respondents diagnosed at stage II and stage III spent 24.5 and 54.8 million VND ($1057-2365) more, respectively, for D&T of BC compared to those diagnosed at stage 0/I. Likewise, respondents with a graduate degree had a total cost of treatment at 62.4 million VND ($2692) higher than those who completed at least secondary education. The completed-data estimations for total cost (pooled-estimations from 20 imputation sets) were 61.8 million VND ($2667) with a wide range from 9.6 to 149.9 million VND ($414–6468).

**Table 2:** Generalised linear model (GLM) analysis of associated factors with total cost of breast cancer care

	Coefficient	95% CI
Stage of cancer at diagnosis^a^		
Stage 0/I^ref^	1.0	–
Stage II	**24.5**	1.2–47.9*
Stage III	**54.8**	20.0–89.6*
Don’t know/Don’t remember	26.3	− 31.4 – 84.1
Household monthly income (in Vietnamese Dong - VND)		
≤ 3,000,000 VND ($129)^ref^	1.0	–
3,000,001–6,000,000 VND ($130–259)	8.0	−40.4 – 56.5
6,000,001–9,000,000 VND ($260–389)	− 11.4	− 44.0 – 21.2
9,000,001–12,000,000 VND ($390–519)	9.0	−22.5 – 40.4
> 12,000,000 VND ($519)	6.5	−22.1 – 35.1
Reimbursement rate of social health insurance		
80%^ref^	1.0	–
95%	−8.2	−33.3 – 16.9
100%	10.3	−19.0 – 39.7
Age	−0.3	−1.0 – 0.4
Education level		
Completed at least secondary education	1.0	–
Completed high school education	15.0	−20.2 – 50.1
Completed undergraduate	6.4	−21.4 – 34.2
Completed graduate	**62.4**	23.6–101.2*

*re*f Reference group | $: United States Dollar (USD) | VND: Vietnamese Dong (currency of Viet Nam)

Exchange rate in November 2020: 1 USD = 23,176 VND

^a^No respondents were diagnosed at stage IV of breast cancer

* *p* < 0.05

## Discussion

The results of this study indicate that BC patients in Vietnam incurred significant OOP costs and the cost of BC treatment was driven by the use of therapies and presentation stage at diagnosis. It is important to note that the analysis was based on a sample in which the proportion of patients diagnosed at early stage (0/I/II) was much higher than the national average of 50.5% [29] though the age of respondents was consistent with other studies [30, 31].

The mean total OOP cost of D&T for BC in Vietnam, excluding the cost of targeted therapy, was 61.8 million VND ($2667) (range: 9.6 to149.9 million VND ($414–6468) - approximately five times higher than the mean cost of 11.7 million VND ($633) reported in the only other study about costs of BC in Vietnam [11]. This difference in estimates may be due to several reasons. The former study used 2001–2006 patient data when medical equipment and medications were less advanced and their use was more restricted. Secondly, costs were calculated from the public payer’s perspective using unit costs which, in Vietnam, are much lower than the real cost of the resources used due to underestimation of health workforce remuneration and capital depreciation [11, 32]. From the patient’s perspective, our study reported the OOP cost which reflected the cost borne by patients and reveal the financial burden they faced. For example, the unit cost of mastectomy regulated by the Ministry of Health in 2018 was 4.7 million VND ($204) [24]. In our study, the cost borne by the patients were reported at 18.4 million VND ($794) which was almost four times higher.

Although the costs reported in this study were much higher than the previous study, it seems much lower than the neighbouring country China or high-income countries like the US (3 and 5–15 times lower, respectively) [33–35]. Comparison of treatment costs in Vietnam with other low- and middle-income countries is not feasible due to the absence of studies with comparable methods (i.e., time horizons of costs, source of data, costing perspective). Moreover, comparison should be used with great caution as between-country differences in treatment costs are likely to be influenced by the variation in treatment guidelines, availability of treatment therapy, stage at diagnosis, and access to health services [13].

The wide range of initial treatment cost is similar with the previous study [11]. Variation in costs may be influenced by types of health services related to BC D&T and the utilisation of SHI for each service. All respondents in the study had SHI and the majority (82%) had SHI with 80% reimbursement rate (co-payment = 20%). However, possession of SHI did not mean respondents could or wanted to utilise their SHI for every service related to BC D&T. There were two possible scenarios: 1) patients chose intentionally not to use SHI and opted to access ‘service- on-demand’ at public hospitals or services at private hospitals (patients pay 100% OOP) which tended to be viewed as providing better quality healthcare; 2) patients could not use SHI for services which were not covered. The lowest utilisation level of SHI was for breast reconstruction surgery and mastectomy (80–88%). With ‘service-on-demand’, patients receive premium service with privileges (i.e., the right to choose a surgeon, time of operation, private hospital room, and level of care) which might partly explain why patients chose this option instead of using standard service covered by the SHI. Further research on how the utilisation of SHI affects the various aspects of treatment (e.g., waiting time, length of treatment, quality, and cost) is needed to generate appropriate policy recommendations for better treatment outcomes.

The analysis showed the overwhelming dominance of targeted therapy cost in relation to total cost of care for patient with HER2+ (who are eligible for targeted therapy). The mean cost of targeted therapy was 649.5 million VND ($28,025) - tenfold higher than the total cost of all other healthcare services related to BC D&T. The share of targeted therapy would be 86% if it were included in the total cost. A study in Portugal found that systemic therapy (targeted therapy + chemotherapy) accounted for 69.2% of the total treatment cost of HER2+ patients which, in turn, was four times higher than patients with other BC subtype [36]. Although 100% of respondents used SHI for targeted therapy, the cost borne by patients was still much higher compared to other service costs due to the high-cost nature of targeted therapy and low SHI reimbursement rate (e.g., SHI covered 60% of the cost of Trastuzumab meaning patients paid 40% of the total cost by OOP plus 20% co-payment of the part covered by SHI [24]). Targeted therapy for HER2+ patients does not tend to be prescribed in Vietnam when it is known that patients cannot afford the treatment (personal communication). The effect of cost on doctor’s prescribing behaviour and patient treatment decisions were documented elsewhere [37–39] and need further research in Vietnam. Polices involving higher SHI reimbursement rate for targeted therapy and/or an OOP maximum (a cap on the amount of money that a patient pays for covered health services plan per year) is likely to impact positively on access by patients to appropriate BC treatment.

When targeted therapy was excluded from total cost, the largest share of cost belonged to chemotherapy (33%) with a mean cost of 36.5 million VND ($1575). Diagnosis accounted for the smallest share in total cost (2%) with a mean cost of 2.6 million VND ($112). This cost composition is consistent with the previously noted study in Vietnam [11]. Cost of diagnosis contained the highest number of extreme outliers. The maximum value with and without outliers was 824 million VND ($35,554) and 17 million VND ($734), respectively. The outliers likely reflect patients having to go through multiple tests at different hospitals before reaching the definitive diagnosis of BC [21].

Multivariate analysis revealed that later stage at diagnosis and higher education level of respondents were associated with higher total OOP cost of D&T for BC. Age, household monthly income, and reimbursement rate of SHI were not significantly associated with costs, similar to the previous study in Vietnam [11]. The mean OOP costs of BC D&T at stage II and III were, respectively, 66 and 148% higher than stage 0/I. The trend is similar though higher than the pooled result from a systematic review of global treatment costs of BC by stage in which the rate was 32 and 95% respectively [13]. Higher costs borne by patients in stage II/III is understandable as their utilisation of mastectomy, chemotherapy, and radiotherapy, which accounted for nearly two-third of the total cost, was significantly higher than stage 0/I patients. The higher costs of later cancer stage emphasise the importance of early detection through screening programme. Policies that help downstaging BC at diagnosis will lessen the costs of treatment borne by the patients and their financial toxicity as well as increase the access to care and outcomes of treatment.

This study provides updated and detailed OOP costs for BC D&T in Vietnam as well as associated factors, using patient-level data. This is only the second study on the subject about costs related to BC and the first study in the country that analysed data from the patient’s perspective. Apart from complementing the previous study which looked at costs from public payer’s perspective, the study provides novel and valuable insights that will facilitate the evaluation of novel therapies in terms of cost-effectiveness in Vietnam including early detection. In turn, the results will help decision-making by policymakers regarding health system financing and service delivery. Although the sample contains a higher proportion of individuals with higher education level and from urban areas if compare with the national data, the poor and near poor were well represented in the sample. Geographical wise, the sample consisted of patients who were treated in main public hospitals from all three regions of Vietnam (the North, the Central, and the South). Thus, study’s results have good generalisability.

It is important to note that the study has some limitations. All costs were self-reported by respondents and were subject to recall bias though by cross-checking information with service price lists in hospitals or regulated by the government and market price of drugs, no unreasonable or inconsistent data were flagged. The practicality of cost data was checked by the cancer survivors in the study advisory board as well. Due to risk of recall bias and difficulties in measurement, we could not collect the costs of follow-up treatment; direct non-medical costs (i.e., transportation, meal, accommodation) and indirect costs (i.e., lost income, premature death) were also not gathered. Future studies should try to include all these costs in the analysis to provide a more complete view on economic impact of BC treatment in Vietnam. There were no survivors diagnosed with stage IV in the sample and this fact affected cost comparisons. We applied a multiple imputation technique to deal with missing data. Although the method used for imputation was technically sound and several pre-cautionary steps were taken to obtain the best reliable imputed values, there remains a possibility of bias.

## Conclusion

The average OOP for D&T of BC in Vietnam was 61.8 million VND ($2667) which is substantially higher than previous estimates based on costs incurred by the government and indicates the considerable financial burden associated with BC to the patients and their families. The costs generally increase with the advancement of the stage of cancer at diagnosis. Stage as a significant driver of cost suggests there exists scope for policies aim at early detection to reduce both the health and economic impacts of BC.

## Supplementary Information

**Additional file 1.** Related questions (extracted from the questionnaire) were used to estimate the cost of breast cancer diagnosis and initial treatment.

## Data Availability

All data generated or analysed during this study are included in this published article.

## Abbreviations

BC: Breast cancer
D&T: Diagnosis and initial treatment
GLM: Generalised linear model
SHI: Social health insurance
IQR: Interquartile range
OOP: Out-of-pocket payment
PMM: Predictive mean matching
Q1: First quartile
Q3: Third quartile
SD: Standard deviation
US: The United States of America
VND: Vietnamese Dong

## References

[1] Van Minh H, Kim Phuong NT, Saksena P, James CD, Xu K (2013). Financial burden of household out-of pocket health expenditure in Viet Nam: findings from the National Living Standard Survey 2002-2010. Soc Sci Med.

[2] NTK T, Tuan PL, Long NH, Thanh PT, Bales S, Vietnam Ministry of Health, Health Partnership Group (2013). Join Annual Health Review 2013: towards Universal Health Coverage.

[3] Out-of-pocket expenditure (% of current health expenditure). 2020. Available from: https://data.worldbank.org/indicator/SH.XPD.OOPC.CH.ZS. [Cited Dec 12, 2020]

[4] Thu Thuong NT, Van Den Berg Y, Huy TQ, Tai DA, Anh BNH (2021). Determinants of catastrophic health expenditure in Vietnam. Int J Health Plann Manag.

[5] Hoang VM, Pham CP, Vu QM, Ngo TT, Tran DH, Bui D, et al. Household financial burden and poverty impacts of cancer treatment in Vietnam. Biomed Res Int. 2017;2017:9350147. 10.1155/2017/9350147. Epub 2017 Aug 21. 10.1155/2017/9350147 PMC5585616 28904976

[6] Jenkins C, Minh LN, Anh TT, Ngan TT, Tuan NT, Giang KB (2018). Breast cancer services in Vietnam: a scoping review. Glob Health Action.

[7] Ngan TT, Van Minh H, Donnelly M, O’Neill C (2021). Financial toxicity due to breast cancer treatment in low- and middle-income countries: evidence from Vietnam. Support Care Cancer.

[8] Gibson TB, Ozminkowski RJ, Goetzel RZ (2005). The effects of prescription drug cost sharing: a review of the evidence. Am J Manag Care.

[9] Eaddy MT, Cook CL, O'Day K, Burch SP, Cantrell CR (2012). How patient cost-sharing trends affect adherence and outcomes: a literature review. P T.

[10] Tai B-WB, Bae YH, Le QA (2016). A systematic review of health economic evaluation studies using the patient’s perspective. Value Health.

[11] Lan NH, Laohasiriwong W, Stewart JF, Tung ND, Coyte PC (2013). Cost of treatment for breast cancer in Central Vietnam. Glob Health Action.

[12] Ministry of Health. In: Son NT, Khue LN, Khoa MT, editors. Guidelines for diagnosis and treatment of breast cancer. Hanoi: Medical Publishing House; 2020.

[13] Sun L, Legood R, Dos-Santos-Silva I, Gaiha SM, Sadique Z (2018). Global treatment costs of breast cancer by stage: a systematic review. Plos One.

[14] Tien TV, Phuong HT, Mathauer I, Phuong NTK. A health financing review of Vietnam with focus on social health insurance. Hanoi: World Health Organization; 2011.

[15] Cheng T-M (2014). Vietnam’s health care system emphasizes prevention and pursues universal coverage. Health Aff.

[16] Amendements to the Law on health insurance, Pub. L. No. 46/2014/QH13 (June 13, 2014).

[17] Lee H-Y, Oh J, Hoang VM, Moon JR, Subramanian SV (2019). Use of high-level health facilities and catastrophic expenditure in Vietnam: can health insurance moderate this relationship?. BMC Health Serv Res.

[18] Thanh NX, Tran BX, Waye A, Harstall C, Lindholm L (2014). Socialization of health care in Vietnam: what is it and what are its pros and cons?. Value Health Reg Issues.

[19] Ngan TT, Mai VQ, Minh HV, Donnelly M, O'Neill C. Health-related quality of life among breast cancer patients compared to cancer survivors and age-matched women in the general population in Vietnam. Qual Life Res. 2021.10.1007/s11136-021-02997-w. Epub 2021 Sep 20 10.1007/s11136-021-02997-w PMC8921138 34541610

[20] World Economic Outlook Database October 2018. 2018 . Available from: https://www.imf.org/external/pubs/ft/weo/2019/01/weodata/index.aspx. [Cited 23 April 2020].

[21] Jenkins C, Ngan TT, Ngoc NB, Hien HT, Anh NH, Lohfeld L (2020). Experiences of accessing and using breast cancer services in Vietnam: a descriptive qualitative study. BMJ Open.

[22] General Statistics Office (2016). Result of the Viet Nam household living standards survey 2016.

[23] National Institute of Standards and Technology (NIST) and SEMATECH. What are outliers in the data? 2013. In: e-handbook of statistical methods. USA: 10.18434/M32189.

[24] Circular on the Promulgation of List of Pharmaceuticals, Biological Drugs, Biochemic Drugs, and Marking Substances Covered by Health Insurance, No.: 30/2018/TT-BYT. Ministry of Health (October 30, 2018).

[25] StataCorp (2013). STATA Multiple-imputation Reference Manual - Release 13.

[26] Buuren Sv. Flexible imputation of missing data. Boca Raton: CRC Press, Taylor & Francis Group; 2012.

[27] Rubin DB. Multiple imputation for nonresponse in surveys. New York: John Wiley & Sons, Inc.; 1987.

[28] Kleinke K (2018). Multiple imputation by predictive mean matching when sample size is small. Methodology.

[29] Thuan TV, Anh PT, Tu DV, TTT H. Cancer control in Vietnam: where are we. Cancer care in emerging health systems. Cancer Control. 2016; Available from: http://www.cancercontrol.info/cc2016/cancer-control-in-vietnam-where-we-are/.

[30] Lan NH, Laohasiriwong W, Stewart JF (2013). Survival probability and prognostic factors for breast cancer patients in Vietnam. Glob Health Action.

[31] Trieu PDY, Mello-Thoms C, Brennan PC (2015). Female breast cancer in Vietnam: a comparison across Asian specific regions. Cancer Biol Med.

[32] Flessa S, Dung NT (2004). Costing of services of Vietnamese hospitals: identifying costs in one central, two provincial and two district hospitals using a standard methodology. Int J Health Plann Manag.

[33] Tollestrup K, Frost FJ, Stidley CA, Bedrick E, McMillan G, Kunde T (2001). The excess costs of breast cancer health care in Hispanic and non-Hispanic female members of a managed care organization. Breast Cancer Res Treat.

[34] Allaire BT, Ekwueme DU, Poehler D, Thomas CC, Guy GP, Subramanian S (2017). Breast cancer treatment costs in younger, privately insured women. Breast Cancer Res Treat.

[35] Liao XZ, Shi JF, Liu JS, Huang HY, Guo LW, Zhu XY (2018). Medical and non-medical expenditure for breast cancer diagnosis and treatment in China: a multicenter cross-sectional study. Asia Pac J Clin Oncol.

[36] Brandão M, Morais S, Lopes-Conceição L, Fontes F, Araújo N, Dias T (2020). Healthcare use and costs in early breast cancer: a patient-level data analysis according to stage and breast cancer subtype. ESMO Open.

[37] Greenup RA, Rushing C, Fish L, Campbell BM, Tolnitch L, Hyslop T (2019). Financial costs and burden related to decisions for breast Cancer surgery. J Oncol Pract.

[38] Eraso Y (2019). Factors influencing oncologists’ prescribing hormonal therapy in women with breast cancer: a qualitative study in Córdoba, Argentina. Int J Equity Health.

[39] Salampessy BH, Alblas MM, Portrait FRM, Koolman X, van der Hijden EJE (2018). The effect of cost-sharing design characteristics on use of health care recommended by the treating physician; a discrete choice experiment. BMC Health Serv Res.

