# Supplementary material for: Costs of breast cancer treatment incurred by women in Vietnam
Source: BMC Public Health. 2022 Jan 10;22:61. doi: 10.1186/s12889-021-12448-3 (PMC8750856; doi:10.1186/s12889-021-12448-3)
Supplement: Supplementary file 1 — Additional file 1. Related questions (extracted from the questionnaire) were used to estimate the cost of breast cancer diagnosis and initial treatment. [file 12889_2021_12448_MOESM1_ESM.docx]

**Related questions (extracted from the questionnaire^[[1]](#footnote-1)^) were used to estimate the cost of breast cancer diagnosis and initial treatment**

| 1. **What types of following health services have you received or receiving?**   *Ask for each row. If respondents answer “No” for B6, skip B7 and B8 of that row and move to the next row.* | | | **B7. If yes, what were the costs you paid for that service?** | **B8. Did the health insurance pay for any part of the service’s cost?** | |
| --- | --- | --- | --- | --- | --- |
|  | **Yes** | **No** |  | **Yes** | **No** |
| Health services relate to examination and diagnosis (e.g., *clinical breast examination, ultrasound, mammography, lab test (e.g.: blood test, etc.), biopsy etc.)* |  |  |  |  |  |
| Lumpectomy *(breast-conserving surgery, only the part of the breast containing the cancer is removed)* |  |  |  |  |  |
| Mastectomy *(complete removal of all breast gland tissue)* |  |  |  |  |  |
| Breast reconstruction surgery *(using an implant or your own tissue)* |  |  |  |  |  |
| Chemotherapy |  |  |  |  |  |
| Radiotherapy |  |  |  |  |  |
| Targeted therapy |  |  |  |  |  |
| Other (please specify) |  |  |  |  |  |

1. This version of the questionnaire was the one used in hospital-based survey with instruction for interviewers (online version has the same content but different instruction to facilitate the self-administrative responses from respondents). The questionnaire was first designed in English. Data collection used questionnaire in Vietnamese. [↑](#footnote-ref-1)
